# Investigating Factors Contributing to a Prolonged Patient Stay (>>72 Hours) in the Mental Health Crisis Assessment Centre (MHCAS).

**DOI:** 10.1192/bjo.2026.11629

**Published:** 2026-06-30

**Authors:** Zi Rui Ong, Donghyun Kim, Mehtab Rahman

**Affiliations:** 1 Imperial College London, London, United Kingdom; 2 Central and North West London NHS Foundation Trust, London, United Kingdom

## Abstract

**Aims:**

MHCAS is a new NHS service that manages patients with an acute mental health crisis, ensuring they are turned around quickly and safely, relieving pressure on hospital wards.

A prolonged patient stay in MHCAS indicates high-risk patients who cannot be discharged safely within 72 hours. Identifying factors associated with delayed discharge can help us characterise high-risk patients and inform safe and efficient patient management strategies. This information can be useful for future MHCAS services across the NHS, aiding in service development and resource allocation.

We aim to identify the key reasons for delayed patient discharge (>72 hours) in MHCAS.

**Methods:**

A retrospective data analysis of 96 admissions lasting >72 hours between July and September 2025 was conducted. The main factors examined were discharge destination, borough of origin, diagnoses, and suicidality. The cumulative hours were analysed by discharge destinations as well as the diagnoses based on ICD-10 and averaged per case.

**Results:**

For discharge location, 19.79% were discharged to temporary residence, contributing 141.10h per patient; 42.71% to wards, contributing 139.53h per patient; and 35.42% to their usual place of residence, contributing 120.87h per patient in MHCAS.

For diagnoses, patients with Schizophrenia Spectrum and Psychotic Disorders contributed the longest average stay per admission (138.84h), followed by Trauma and Stress related disorders (133.33h) and Bipolar Spectrum Disorders (128.83h). Schizophrenia Spectrum and Psychotic Disorders were the most common diagnoses (23%), followed by Depressive Disorders (20%) and Anxiety and Somatoform Disorders (12%).

The majority of patients came from CNWL boroughs (88.37%), and Westminster was the biggest contributing borough (30%).

Most patients with prolonged stays were admitted via emergency pathways, primarily A&E or the Home Treatment Team (83%), indicating that these admissions largely reflect high-risk presentations.

Regarding suicidality, 39 patients reported suicidal ideation, 41 did not, and 16 cases were undocumented. Suicidal patients spent 8.52 hours more in MHCAS on average.

**Conclusion:**

Prolonged stays in MHCAS are influenced by multiple factors. Ward admissions and temporary accommodation contributed significantly, showing that delays in beds availability or social services played a key role. Patients with more severe diagnoses, such as schizophrenia, are high-risk, therefore requiring more prolonged care. Information transfer from non-CNWL boroughs could also have resulted in delays in care planning.

Contrary to expectations, suicidal ideation was only associated with a modest increase in length of stay, indicating suicidality alone is not a primary driver.

